# Safety and immunogenicity of an inactivated recombinant Newcastle disease virus vaccine expressing SARS-CoV-2 spike: A randomised, comparator-controlled, phase 2 trial

**DOI:** 10.1016/j.vaccine.2024.126542

**Published:** 2025-01-12

**Authors:** Vu Dinh Thiem, Dang Duc Anh, Vu Hai Ha, Nguyen Van Thom, Tran Cong Thang, Jose Mateus, Juan Manuel Carreño, Rama Raghunandan, Nguyen Mai Huong, Laina D. Mercer, Jorge Flores, E. Alexandar Escarrega, Ariel Raskin, Duong Huu Thai, Le Van Be, Alessandro Sette, Bruce L. Innis, Florian Krammer, Daniela Weiskopf

**Affiliations:** a National Institute of Hygiene and Epidemiology, 1 Yersin Street, Hai Ba Trung District, Hanoi, Viet Nam; b Center for Disease Control, Thai Binh Province, 10 Hoàng Công Chất street, Quang Trung ward, Thai Binh, Viet Nam; c PATH Vietnam, Hanoi Towers, 49 Hai Ba Trung Street, Hoan Kiem District, Hanoi, Viet Nam; d Center for Infectious Disease and Vaccine Research, La Jolla Institute for Immunology (LJI), La Jolla, CA, USA; e Department of Microbiology, Icahn School of Medicine at Mount Sinai New York, New York, NY, USA; f Center for Vaccine Research and Pandemic Preparedness (C-VaRPP) at Mount Sinai New York, New York, NY, USA; g Center for Vaccine Innovation and Access, PATH, 2201 Westlake Avenue, Suite 200, Seattle, WA 98121, USA; h Institute of Vaccines and Medical Biologicals, 9 Pasteur, Xuong Huan, Nha Trang City, Khanh Hoa, Viet Nam; i Department of Medicine, University of California, San Diego, (UCSD), La Jolla, CA, 92037, USA; j Department of Pathology, Molecular and Cell-Based Medicine, Icahn School of Medicine at Mount Sinai, NY, New York, USA

## Abstract

Abstract

Production of affordable coronavirus disease 2019 (COVID-19) vaccines in low- and lower-middle-income countries is needed. NDV-HXP-S is an inactivated egg-based recombinant Newcastle disease virus vaccine expressing the spike protein of severe acute respiratory syndrome coronavirus 2 (SARS-CoV-2). A public sector manufacturer in Vietnam assessed the immunogenicity of NDV-HXP-S (COVIVAC) relative to an authorized vaccine.

This phase 2 stage of a randomised, observer-blind, controlled, phase 1/2 trial was conducted at three community health centers in Thai Binh Province, Vietnam. Healthy males and non-pregnant females, 18 years of age and older, were eligible. Participants were randomised by age (18–59, ≥60 years) to receive one of three treatments by intramuscular injection twice, 28 days apart: COVIVAC at 3 μg or 6 μg, or AstraZeneca COVID-19 vaccine VAXZEVRIA™. Participants and personnel assessing outcomes were masked to treatment. The vaccine dose was selected based on Phase 1 results. A 6 μg dose was chosen to explore the immunogenicity gain over the 3-μg dose.

The study's aim is to evaluate the safety and immunogenicity of COVIVAC at two dose levels compared to VAXZEVRIA, the most commonly used COVID-19 vaccine in Vietnam. The main outcome was the induction of 50% neutralising antibody titers against vaccine-homologous pseudotyped virus 14 days (day 43) and 6 months (day 197) after the second vaccination by age group. The primary immunogenicity and safety analyses included all participants who received one dose of the vaccine. ClinicalTrials.govNCT05940194.

During August 10–23, 2021, 737 individuals were screened, and 374 were randomised (124–125 per group); all subjects received vaccine dose one and all but three received doses two four weeks later. Subjects 18–59 years of age achieved the following geometric mean titers of PNA 14 days after vaccine dose two: 153⋅28 (95 % CI 124·2–189⋅15) for COVIVAC 3 μg, 176⋅2 (95 % CI 141⋅45–220.27) for COVIVAC 6 μg, and 99⋅92(95 % CI 80.80–123⋅56) for VAXZEVRIA. Subjects ≥60 years of age also achieved potent geometric mean titers of PNA at the same timepoint: 183⋅57 (95 % CI 133.4–252⋅61) for COVIVAC 3 μg, 257⋅87 (95 % CI 181⋅6–367⋅18) for COVIVAC 6 μg, and 79⋅49(95 % CI 55⋅68–113⋅4) for VAXZEVRIA.

On day 43, the geometric mean fold rise of 50 % neutralising antibody titers for subjects age 18–59 years was 31·20 (COVIVAC 3 μg *N* = 82, 95 % CI 25·14–38·74), 35·80 (COVIVAC 6 μg; *N* = 83, 95 % CI 29·03–44·15), 18·85 (VAXZEVRIA; N = 82, 95 % CI 15·10–23·54), and for subjects age ≥ 60 years was 37·27 (COVIVAC 3 μg; *N* = 42, 95 % CI 27·43–50·63), 50·10 (COVIVAC 6 μg; *N* = 40, 95 % CI 35·46–70·76), 16·11 (VAXZEVRIA; N = 40, 95 % CI 11·73–22·13). Among subjects seronegative for anti-S IgG at baseline, the day 43 geometric mean titer ratio of neutralising antibody (COVIVC 6 μg/VAXZEVRIA) was 1·77 (95 % CI 1·30–2·40) for subjects age 18–59 years and 3·24 (95 % CI 1·98–5·32) for subjects age ≥ 60 years. On day 197, the age-specific ratios were 1·11 (95 % CI 0·51–2·43) and 2·32 (0·69–7·85). Vaccines were well tolerated; reactogenicity was predominantly mild and transient. The percentage of subjects with unsolicited adverse events (AEs) during 28 days after vaccinations was similar among treatments (COVIVAC 3 μg 29·0 %, COVIVAC 6 μg 23·2 %, VAXZEVRIA 31·2 %); no vaccine-related AE was reported. Considering that induction of neutralising antibodies against SARS-CoV-2 has been correlated with the efficacy of COVID-19 vaccines, including VAXZEVRIA, our results suggest that vaccination with COVIVAC may afford clinical benefit matching or exceeding that of the VAXZEVRIA vaccine.

ClinicalTrials.govNCT05940194

Keywords

SARS-CoV-2

COVID-19

Newcastle disease virus

Egg-based vaccine

Phase II clinical trial

## 1 Introduction

The coronavirus disease 2019 (COVID-19) pandemic has resulted in millions of deaths, burdened healthcare systems globally, and exposed vaccine access inequities worldwide. A systematic study to assess the impact of delayed supply of COVID-19 vaccines indicated that only 25 % of the population in low- and lower-middle-income countries received at least one dose of vaccine as of October 2022 [1]. Ensuring an adequate supply of COVID-19 vaccines for low- and lower-middle-income countries (LMICs), which constitute 85 % of the global population, is essential.

As of March 2023, Vietnam's Ministry of Health recorded 11,525,408 COVID-19 cases, ranking thirteenth in the amount of cases among 230 countries and territories worldwide [2]. Although imported vaccines and infection-induced immunity have reduced the risk of disease, the threat from new viral variants and the potential need for vaccinating elderly adults and other at-risk individuals annually highlight the value to Vietnam of access to domestically produced COVID-19 vaccines as a sustainable asset.

The rapid rollout of COVID-19 vaccines saved millions of lives globally [3]. By inducing potent severe acute respiratory syndrome coronavirus 2 (SARS-CoV-2) antibodies, COVID-19 vaccines reduce the risk of severe disease, with the level of antibodies induced correlated with vaccine efficacy [4,5]. However, the emergence of Omicron sub-lineage variants with increased transmissibility and escape from pre-existing neutralising antibodies emphasizes the importance of confirming that new COVID-19 vaccine candidates also induce cellular immunity [6].

PATH and the Icahn School of Medicine at Mount Sinai collaborated with Vietnam's Institute of Vaccines and Medical Biologicals (IVAC), a manufacturer of egg-based inactivated influenza vaccines, to develop an egg-based inactivated Newcastle disease virus vaccine expressing a six-proline prefusion-stabilized SARS-CoV-2 spike (NDV-HXP-S COVID-19 vaccine, also known as COVIVAC) [7]. In a phase 1 trial (NCT04830800), COVIVAC administered twice 28 days apart had an acceptable safety and immunogenicity profile in healthy adults 18–59 [8]. For the next stage of clinical development, IVAC sponsored a phase 2 trial in which the safety and immunogenicity of COVIVAC at two dosage levels, in adults with stable health including individuals ≥60 years of age, was contrasted with AstraZeneca's adenovirus vectored COVID-19 vaccine (VAXZEVRIA) [9] then the authorized pandemic vaccine most commonly administered in Vietnam. The study aimed to demonstrate that COVIVAC induced a superior neutralising antibody response to vaccine-homologous SARS-CoV-2 relative to VAXZEVRIA. The study also aimed to explore the activation of SARS-CoV-2 spike-specific T cells by COVIVAC versus VAXZEVRIA. This report provides the results of that clinical trial, including the induction of virus neutralising antibodies against pseudotyped and wild-type (live virus) vaccine-homologous SARS-CoV-2 and virus-specific T-cell activation.

## 2 Methods

### 2.1 Study design and participants

This phase 2 stage (NCT05940194) of a randomised, observer-blind, controlled, phase 1/2 trial was conducted at three community health centers within the Vu Thu District Health Center catchment, Thai Binh Province, Vietnam. Investigators from Vietnam's National Institute of Hygiene and Epidemiology collaborated with staff of the community health centers, district health center, district hospital, and the Provincial Center for Disease Control to perform the study. Participants were recruited following community outreach. Males and non-pregnant females (sex or gender was self-reported) with stable health, 18 years of age and older, with body mass index 17 to 40 kg/m^2^, with no history of confirmed COVID-19 or infection with human immunodeficiency virus, were eligible to participate. A negative urinary pregnancy test was required of women with reproductive capacity before administering each study vaccine dose. Complete eligibility criteria are described in the trial protocol provided in the supplementary material.

There were two exclusion criteria in the protocol regarding the vaccination history as follows: (1) history of administration of any non-study vaccine within 28 days prior to administration of study vaccine or planned vaccination within 3 months after enrolment (although receipt of any COVID-19 vaccine that is licensed or granted Emergency Use Authorization in Vietnam during the course of study participation won't be exclusionary if administered after Visit 5); and (2) previous receipt of investigational vaccine for SARS or MERS, or any investigational or licensed vaccine that may have an impact on interpretation of the trial results.

Written informed consent was obtained from all participants. The trial complied with the Declaration of Helsinki and Good Clinical Practice. This study was jointly approved by the Institutional Review Board of the Vietnam National Institute of Hygiene and Epidemiology and the Independent Ethics Committee of the Vietnam Ministry of Health ref. no. 1407/QD-BYT.

### 2.2 Randomisation and masking

Subjects (*N* = 374) were randomly allocated to one of three equal groups (COVIVAC 3 μg, COVIVAC 6 μg, or the comparator VAXZEVRIA) using a computer-generated randomisation sequence prepared by an unblinded statistician. Randomisation was age-stratified, with approximately one-third of subjects aged ≥60 years. An unmasked pharmacist dispensed each treatment according to the randomisation sequence to an unmasked vaccinator. All participants and study personnel, besides the unmasked pharmacy team and vaccinators, were masked for treatment.

### 2.3 Procedures

The recombinant NDV-HXP-S vaccine expressing wild type Wuhan strain S protein (COVIVAC) was manufactured according to current Good Manufacturing Practice (GMP) by IVAC in their Influenza Vaccine Plant (Nha Trang, Vietnam), as previously described [8]. The adenovirus vectored vaccine from AstraZeneca (ChAdOx1; VAXZEVRIA), used as a comparator vaccine, was sourced from the Ministry of Health. Unmasked vaccinators administered study treatments by intramuscular injection of 0·5 mL on study days 1 and 29. Subjects were observed in the clinic for 30 min after each vaccination. Blood samples were drawn for immunogenicity endpoints before vaccination on days 1 (first dose), 43 (14 days post dose two), and 197 (6 months post dose two). Subjects randomly allocated to a cell-mediated immunity subset (*N* = 12 per treatment group) had additional blood collected on days 1 and 43 to isolate peripheral blood mononuclear cells (PBMCs); these were stored in liquid nitrogen until analysed. Solicited injection site reactions (pain/tenderness, swelling/induration, erythema) and systemic symptoms (headache, fatigue, malaise, myalgia, arthralgia, nausea/vomiting, and fever defined as oral temperature ≥ 38 °C) were recorded by subjects in a diary card for seven days post-vaccination that included intensity, which the investigators then reported. Subjects also recorded unsolicited adverse events (AEs) for 28 days after each vaccine dose and reported them at scheduled clinic visits, whereupon the investigator included these in the study database after interviewing the subjects, grading them for intensity as previously described [8], assessing them for causality, and categorizing them as severe or not. Severe AEs were collected for the duration of the study. A Data Safety Monitoring Board (DSMB) monitored unblinded safety data.

We measured anti-SARS-CoV-2 spike IgG using a validated indirect enzyme-linked immunosorbent assay (ELISA) at Nexelis (Laval, Canada), as described [8]. Concentrations were transformed to binding antibody units per mL (BAU/mL), based on the World Health Organization (WHO) International Standard for anti-SARS-CoV-2 immunoglobulin using a conversion factor determined during assay validation (1/7·9815). The assay's cut-off and lower limit of quantitation (LLOQ) were 6·3 BAU/mL.

We measured serum neutralising activity against the Wuhan-Hu-1 strain of SARS-CoV-2 in a validated pseudotyped virus neutralisation assay (PNA) [8] that assessed particle entry inhibition [10]. The neutralising titer of a serum sample was calculated as the reciprocal serum dilution corresponding to the 50 % neutralisation antibody titer (NT_50_) for that sample; the NT_50_ titers may be transformed to international units per mL (IU/mL), based on the WHO international standard for anti-SARS-CoV-2 immunoglobulin, using a conversion factor determined during assay validation (1/1·872). The assay's cut-off and lower limit of quantitation (LLOQ) were 5·3 IU/mL (10 as the NT_50_ titer value) and 5·9 IU/mL, respectively.

We also measured live virus neutralising activity as a 50 % inhibitory dilution (ID_50_) against a wild-type SARS-CoV-2 isolate (USA-WA1/2020, catalog number NR-52281; BEI Resources) using an assay performed in a biosafety level 3 facility as previously described [11]. Briefly, Vero.E6 cells (20,000 cells/100 μL per well) were seeded onto sterile 96-well cell culture plates a day prior to the neutralisation assay. Sera were serially diluted in minimal essential medium (MEM; Life Technologies) at a 1:10 starting dilution. One thousand (1000) median tissue culture infectious doses (TCID_50_s) of the virus were incubated with diluted sera for 1 h inside a biosafety cabinet. Media from confluent cell monolayers (90 %) was removed, and 120 μL of the virus-serum dilutions were added to the cells for 1 h at 37 °C. The mixture was removed and 100 μL of each corresponding serum dilution was added per well. Additionally, 100 μL of MEM was added to every well. Remdesivir at 10 μM was used as control. Plates were incubated at 37 °C for 48 h, media was removed, and cells were fixed with 150 μL of 10 % formaldehyde (Polysciences) per well. After fixation, cells were permeabilized and stained using the 1C7C7 mAb [11]. The live virus neutralisation assay (LVNA) cutoff (ID_50_) was 1:10.

To assess the breadths of the adaptive immune response, we measured vaccine-induced spike-specific T cells in PBMC samples utilizing a T cell receptor (TCR) dependent activation induced markers (AIM) assay [12,13]. AIM assays have been comprehensively used to compare COVID-19 vaccine-induced T cell responses [14,15]. This assay measures antigen specific T cells based on upregulation of activation markers, irrespective of cytokines [16]. Antigen-specific CD4+ and CD8+ T cells were measured as a percentage of AIM+ T cells+ as described before. [14], [16], [17] Briefly, PBMC were thawed and plated in 96-wells U-bottom plates at 1 × 10^6^ PBMC per well, then blocked at 37 °C for 15 min with 0·5 μg/mL anti-CD40 mAb (Miltenyi Biotec), and fluorescently labeled with chemokine receptor antibodies (anti-CCR6, CXCR5, CXCR3, and CCR7) (see Supplement Table 1 for list of antibodies used). Cells were incubated at 37 °C for 24 h with a spike-specific peptide mega pool (MP; 1 μg/mL); controls were dimethyl sulfoxide (DMSO, an equimolar amount) and phytohaemagglutinin PHA (2·5 μg/mL). The mega pool (MP) approach, previously described, enables simultaneous testing of a large number of epitopes, facilitating the characterization of T cell responses to infectious diseases [13,14]. We stimulated the PBMCs *ex vivo* to evaluate the antigen-specific T cell response against SARS-CoV-2. The spike MP has 253 overlapping peptides spanning the entire sequence of the spike protein [18]. SARS-CoV-2 spike-specific circulating CD4+ T cells and spike-specific circulating CD8+ T cells were measured by surface co-expression of OX40 + CD137+ and CD69 + CD137+, respectively. SARS-CoV-2 spike-specific circulating follicular helper T (cT_FH_) cells were measured as CXCR5 + OX40 + surface CD40L+ and quantified as a percentage of CD4+ T cells after stimulation with spike MP. The samples were acquired on a Cytek Aurora (Cytek Biosciences). The gating strategy is shown in Supplement Fig. 1.

### 2.4 Outcomes

The primary outcomes were safety and induction of neutralising antibodies by COVIVAC, comparing 3 μg to 6 μg and each COVIVAC group to the VAXZEVRIA group. The safety of each treatment was evaluated as the number and severity of solicited injection site and systemic AEs during 7 days after vaccination. Number, severity, and relatedness of unsolicited (spontaneously reported) AEs during 28 days after each vaccination; and occurrence of medically attended AEs, serious AEs, and AEs of special interest throughout the 7-month study period. Induction of neutralising antibody measured by PNA was expressed as a geometric mean titer (GMT) at 14 days post second vaccination, a GMT ratio in subjects seronegative at baseline, a geometric mean fold rise (GMFR), and a percentage of subjects with a ≥ 4-fold increase from baseline regardless of baseline anti-spike IgG seropositivity. A secondary immunogenicity outcome was the induction of anti-spike IgG in binding antibody units (BAU/mL) expressed in the same four parameters used for the neutralising activity. The exploratory immunogenicity outcomes were the induction of neutralising antibodies to wild-type SARS-CoV-2 expressed as a GMT and GMT ratio (COVIVAC/VAXZEVRIA) at 14 days post second vaccination, and the frequency of spike-specific activated T cells.

### 2.5 Statistical analysis

This study (ClinicalTrials.gov NCT05940194) was designed to assess the feasibility of advancing the evaluation of COVIVAC towards emergency use authorization based on non-inferiority or superiority to the comparator. With 375 participants randomised by 1:1:1, the study had >90 % power to demonstrate a lower bound of the 95 % confidence interval (CI) of the GMT ratio greater than 1·0 if the observed ratio (COVIVAC/VAXZEVRIA or COVIVAC 6μg/COVIVAC 3μg) was ≥1·65. These calculations assumed a 10 % loss to follow up and the variability in NT50 was informed by the Phase 1 data. The study also had >95 % power to detect at least one serious or severe adverse event if the underlying rate was ≥2·5 % and power was >80 % to detect differences in AE rates ≥15 %. As a secondary objective, COVIVAC 3 μg and 6 μg were to be compared to AZD1222. Sequential testing of COVIVAC 6 μg versus AZD1222 followed by COVIVAC 3 μg versus AZD1222 (if non-inferiority was shown with 6 μg) were be employed to conserve alpha.

All safety assessments occurred in the treatment-exposed population, according to the treatment received. All treatment group percentages were supplemented with two-sided 95 % confidence intervals (CIs) computed via the Clopper-Pearson method. The immunogenicity analysis presented was performed in the full analysis population that included all subjects randomised for whom any post-vaccination immunogenicity data were available. This population is identical to the per-protocol population at Day 43. Geometric mean antibody responses were reported by treatment and time point, accompanied by 95 % CIs. The analysis of geometric means excluded subjects who were seropositive at baseline (defined by anti-spike IgG > LLOQ as measured by ELISA). GMFRs were calculated relative to baseline using the log difference of the paired samples, with corresponding CIs computed via the t-distribution, utilizing the antilog transformation to present the ratio. The proportions of subjects with GMFRs of NT50 ≥ 4 from baseline were summarized with two-sided 95 % confidence intervals computed via the Clopper-Pearson method. The analysis of immunogenicity relative to baseline included baseline seropositive subjects. The CD4+ T cell responses were summarized via the geometric mean and treatment groups were compared via the Mann-Whitney *U* test. All statistical tests were two-sided with a significance level of 0·05. All statistical analyses were performed using SAS version 9·4.

### 2.6 Role of the funding source

The funders of the study had no role in data collection, data analysis, or writing of the statistical report. IVAC was the clinical trial sponsor and approved the study protocol. IVAC employees contributed as authors by preparing the investigational vaccine, interpreting data, and critically reviewing this report. All authors had full access to all data in the study and accepted responsibility for the decision to submit for publication.

## 3 Results

From August 10 to 23, 2021, 737 individuals were screened and 374 were randomised to three treatment groups (124–125 subjects per group). The study team conducted the informed consent and screening sessions separately from the study vaccination sessions; they invited 737 individuals for informed consent process and screening; among them 177 individuals were not eligible. There were 560 eligible individuals sequentially invited for study vaccination; when 374 subjects were randomised, the protocol-authorized sample size was achieved and randomisation ceased.

**Fig. 1:**
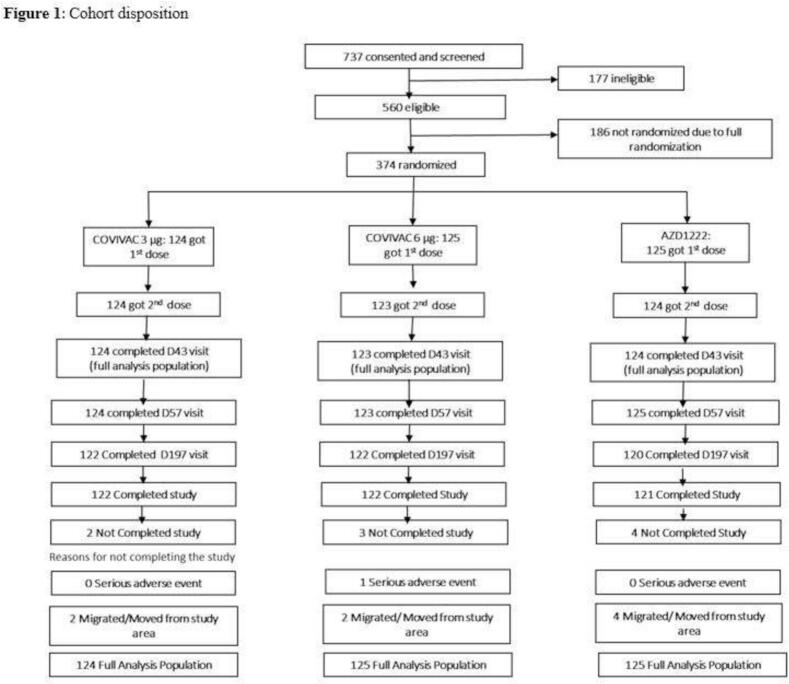
**Cohort disposition:** Disposition of subjects recruited and randomised in Phase 2.

During the trial, all subjects received vaccine dose one and all but three received doses two four weeks later; 365 completed the last study visit on day 197 (Fig. 1). The baseline characteristics are shown by treatment group in Table 1; the exposed population was 49·5 % male, had a mean age of 49 years (range 18–77) and a mean body mass index of 22·29 (range 17·01–31·76).

**Table 1:** Baseline characteristics of the exposed population.

Baseline Characteristics	COVIVAC		VAXZEVRIA
	3 μg (*N* = 124)	6 μg (*N* = 125)	(*N* = 125)
Mean age in years (SD; range)	48·9 (14·82; 18, 75)	48·9 (14·27; 18,77)	49·8 (14·17; 18,74)
Male	64 (51·6 %)	67 (53·6 %)	54 (43·2 %)
Female	60 (48·4 %)	58 (46·4 %)	71 (56·8 %)
Mean body mass index in kg/m2 (SD; range)	22·29 (2·57; 17·01, 31·76)	22·27 (2·67; 17·33, 28·75)	22·24 (2·55; 17·08, 28·88)

Trial participants from all three vaccine groups tolerated the doses with no dose-limiting reactogenicity. Solicited injection site reactogenicity was mostly mild during the seven days after each vaccination (Table 2). Pain or tenderness was the most common injection site symptom recorded, more frequently following dose one than dose two. Post-dose one-injection site pain was reported by 72 % of VAXZEVRIA recipients but by only 46–56 % of COVIVAC recipients. The most common systemic symptoms (Table 2) were fatigue or malaise, headache, and myalgia, reported more frequently following dose one than dose two. Notably, fever (≥38 °C) following dose one occurred in 22·4 % of VAXZEVRIA recipients but in only 0·8 % of COVIVAC recipients. Unsolicited adverse events occurring 28 days after vaccination (Supplement Table 2) were reported by a similar proportion of subjects in each treatment group (23·2–31·2 %); none of these events were judged by the investigator to be treatment-related or led to withdrawal from the trial. Although six serious adverse events were reported during the entire study period (three in each COVIVAC treatment group), none were considered treatment-related (intestinal obstruction, sialadenitis, leukemia, COVID-19, colon cancer, and gastric cancer). The independent DSMB expressed no safety concerns.

**Table 2:** Number of subjects with solicited adverse events during 7 days after vaccination in the safety analysis population.

Reaction		COVIVAC		VAXZEVRIA
		3 μg (N = 124) n (%) (95 % CI*)	6 μg (N = 125) n (%) (95 % CI*)	(N = 125) n (%) (95 % CI*)
Pain/tenderness	Dose 1	57 (46·0 %)	65 (52·0 %)	90 (72·0 %)
		(37·0-55·1)	(42·9-61·0)	(63·3-79·7)
	Dose 2	34 (27·4 %)	48 (39·0 %)	37 (29·8 %)
		(19·8-36·2)	(30·4-48·2)	(22·0-38·7)
Swelling/induration	Dose 1	2 (1·6 %)	1 (0·8 %)	1 (0·8 %)
		(0·2-5·7)	(0·0-4·4)	(0·0-4·4)
	Dose 2	2 (1·6 %)	0 (0 %)	0 (0 %)
		(0·2-5·7)	(0·0-3·0)	(0·0-2·9)
Erythema	Dose 1	0 (0 %)	0 (0 %)	1 (0·8 %)
		(0·0-2·9)	(0·0-2·9)	(0·0-4·4)
	Dose 2	1 (0·8 %)	0 (0 %)	0 (0 %)
		(0·0-4·4)	(0·0-3·0)	(0·0-2·9)
Fever (≥ 38 °C)	Dose 1	1 (0·8 %)	1 (0·8 %)	28 (22·4 %)
		(0·0-4·4)	(0·0-4·4)	(15·4-30·7)
	Dose 2	3 (2·4 %)	3 (2·4 %)	2 (1·6 %)
		(0·5-6·9)	(0·5-7·0)	(0·2-5·7)
Headache	Dose 1	31 (25·0 %)	52 (41·6 %)	63 (50·4 %)
		(17·7-33·6)	(32·9-50·8)	(41·3-59·5)
	Dose 2	28 (22·6 %)	28 (22·8 %)	26 (21·0 %)
		(15·6-31·0)	(15·7-31·2)	(14·2-29·2)
Fatigue/malaise	Dose 1	53 (42·7 %)	59 (47·2 %)	77 (61·6 %)
		(33·9-51·9)	(38·2-56·3)	(52·5-70·2)
	Dose 2	39 (31·5 %)	38 (30·9 %)	40 (32·3 %)
		(23·4-40·4)	(22·9-39·9)	(24·1-41·2)
Myalgia	Dose 1	24 (19·4 %)	26 (20·8 %)	47 (37·6 %)
		(12·8-27·4)	(14·1-29·0)	(29·1-46·7)
	Dose 2	21 (16·9 %)	14 (11·4 %)	23 (18·5 %)
		(10·8-24·7)	(6·4-18·4)	(12·1-26·5)
Arthralgia	Dose 1	23 (18·5 %)	17 (13·6 %)	31 (24·8 %)
		(12·1-26·5)	(8·1-20·9)	(17·5-33·3)
	Dose 2	5 (4·0 %)	9 (7·3 %)	16 (12·9 %)
		(1·3-9·2)	(3·4-13·4)	(7·6-20·1)
Nausea/vomiting	Dose 1	10 (8·1 %)	9 (7·2 %)	13 (10·4 %)
		(3·9-14·3)	(3·3-13·2)	(5·7-17·1)
	Dose 2	2 (1·6 %)	4 (3·3 %)	3 (2·4 %)
		(0·2-5·7)	(0·9-8·1)	(0·5-6·9)

**Fig. 2:**
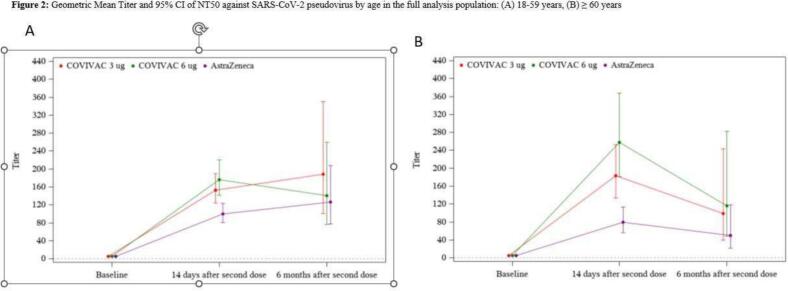
**Geometric mean titer and 95 %** confidence interval **of NT50 against SARS-CoV-2 pseudotyped virus by age and treatment group in the full analysis population:** (a) 18–59 years, (b) ≥ 60 years.

The study's aim is to evaluate the safety and immunogenicity of COVIVAC at two dose levels compared to VAXZEVRIA, the most commonly used COVID-19 vaccine in Vietnam. The main immunogenicity measure was the induction of vaccine-homologous antibodies assessed by PNA 14 days after vaccine dose two. Fig. 2 shows plots of neutralising (PNA) antibody GMT by age and treatment group over time among the 95 % of subjects seronegative at baseline for anti-S IgG and with a valid assay result (see also Supplement Table 3). Responses to COVIVAC were significantly higher than to VAXZEVRIA 14 days after vaccine dose two, although this contrast was not statistically significant six months after vaccine dose two. Note that six months after dose two, GMTs remained well above baseline, with increases observed in two groups among adults 18–59 years of age. Subjects 18–59 years of age achieved the following geometric mean titers of PNA 14 days after vaccine dose two: 153.28 (95 % CI 124·2–189.15) for COVIVAC 3 μg, 176.2 (95 % CI 141.45–220.27) for COVIVAC 6 μg, and 99.92(95 % CI 80.80–123.56) for VAXZEVRIA. Subjects ≥60 years of age also achieved potent geometric mean titers of PNA at the same timepoint: 183.57 (95 % CI 133.4–252.61) for COVIVAC 3 μg, 257.87 (95 % CI 181.6–367.18) for COVIVAC 6 μg, and 79.49(95 % CI 55.68–113.4) for VAXZEVRIA. As the 95 % confidence intervals for GMT substantially overlap between the two age strata for COVIVAC within each tested dosage, the responses are similar (Supplement Table 3).

The GMT in the 3μg group (18–59) on day 14 after two doses was 153 whereas the GMT at 6 months was 188. The modest observed increase in median titer is within the error of the assay method but also may be due to intercurrent infection with SARS-CoV-2 among some subjects.

The percentage of subjects 18–59 years of age mounting a minimum four-fold PNA response to vaccination 14 days after vaccine dose two was 89·0 % (95 % CI 80·2–94·9) for COVIVAC 3 μg, 92·8 % (95 % CI 84·9–97·3) for COVIVAC 6 μg, and 85·4 % (95 % CI 75·8–92·2) for VAXZEVRIA. Equally high PNA response rates were also observed in COVIVAC vaccinees ≥60 years of age (Supplement Table 4). Notably, the magnitude of neutralising antibody induction 14 days after dose two, expressed as a PNA GMFR from baseline, although similar between COVIVAC groups, was greater compared to the VAXZEVRIA group (Table 3) for subjects 18–59 years of age and for subjects ≥60 years of age. The greater peak induction of neutralising antibodies by COVIVAC relative to VAXZEVRIA was also apparent in the GMT ratios (COVIVAC/VAXZEVRIA) for both dose levels with 95 % confidence intervals that excluded 1·00 for both age strata (Supplement Table 5).

**Table 3:** Summary of geometric mean fold rise (GMFR) from baseline of NT_50_ titers against SARS-CoV-2 pseudovirus by age group in the full analysis population.

NT50 MEASURE			COVIVAC		VAXZEVRIA
			3 μg (N = 124)	6 μg (N = 125)	(N = 125)
14 days after the second vaccination (D43)	18–59 yr.	GMFR from baseline (95 % CI)	n = 82	n = 83	n = 82
			31.20 (25·14, 38·74)	35·80 (29·03, 44·15)	18·85 (15·10, 23·54)
	≥ 60 yr.	GMFR from baseline (95 % CI)	n = 42	*n* = 40	n = 40
			37·27 (27·43, 50·63)	50·10 (35·46, 70·76)	16·11 (11·73, 22·13)
6 months after the second vaccination (D197)	18–59 yr.	GMFR from baseline (95 % CI)	*n* = 80	n = 82	n = 80
			39·94 (21·56, 73·98)	32·27 (17·73, 58·72)	22·63 (13·72, 37·34)
	≥ 60 yr.	GMFR from baseline (95 % CI)	n = 40	n = 37	n = 37
			18·31 (7·73, 43·32)	22·52 (9·26, 54·76)	13·36 (5·61, 31·82)

To confirm the observation of COVIVAC's superior peak induction of vaccine-homologous neutralising antibodies, we evaluated the GMT of neutralising antibodies measured by live virus neutralising assay (LVNA) induced by two doses of 3 μg or 6 μg of COVIVAC and compared it with neutralising antibodies induced by VAXZEVRIA. Although the GMTs measured by live virus assays were approximately two thirds less than mean titers measured by pseudotyped virus, the GMT ratios estimated for the two COVIVAC groups relative to the VAXZEVRIA group by either assay were 1.5 to 2.0-fold higher in a dose-dependent manner (Supplement Table 6).

A secondary immunogenicity outcome was the induction of anti-spike IgG in binding antibody units (BAU/mL). By this measure of immunogenicity, VAXZEVRIA induced higher peak concentrations of anti-spike IgG measured by ELISA than did COVIVAC at either dose or for both age strata (Supplement Tables 7–9). For instance, the GMC ratio (COVIVAC 3 μg/VAXZEVRIA) at 14 days after vaccine dose two in subjects 18–59 was 0·38 (95 % CI, 0·29–0·50), and in those ≥60 was 0·47 (95 % CI 0·28–0·78). Six months after dose two, the 95 % CI for the GMC ratios included 1·00. This aligns with earlier observations showing that inactivated NDV-HXP S induces higher neutralising antibody-to-spike binding antibody ratios compared to other vaccine platforms [19].

**Fig. 3:**
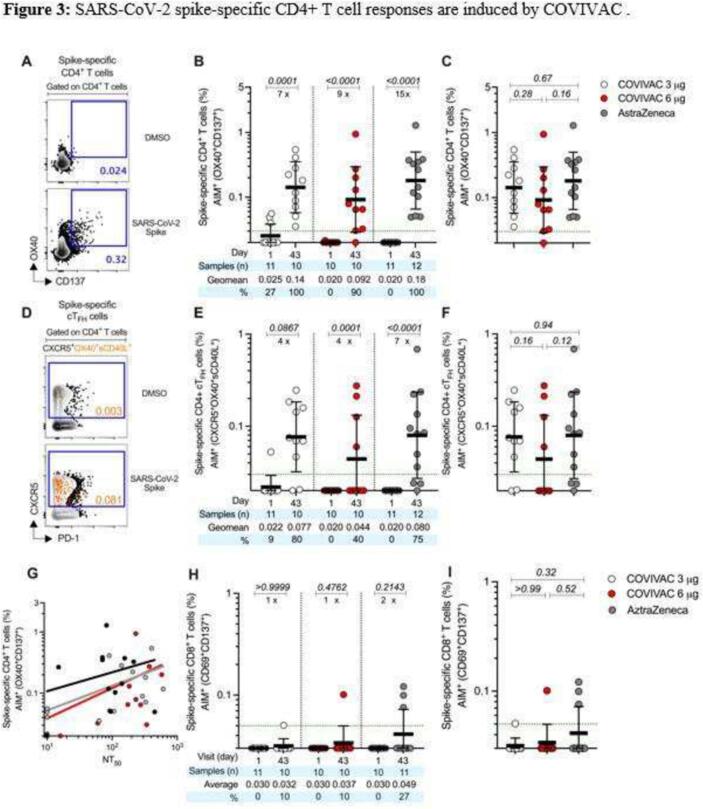
**SARS-CoV-2 spike-specific CD4+ T cell responses are induced by COVIVAC.** (A) FACS example of SARS-CoV-2 spike-specific CD4+ T cells evaluated by the AIM assay after stimulation with spike MP. Spike-specific CD4+ T cells were quantified by AIM (surface OX40 + CD137+) after stimulation with spike peptide megapool (MP). (B) Frequencies of SARS-CoV-2 spike-specific CD4+ T cells induced by COVIVAC at 3 μg and 6 μg and VAXZEVRIA at day 1 (baseline) and at day 43 post-vaccination. (C) Comparison of spike specific CD4+ T cells induced by COVIVAC at 3 μg and 6 μg and VAXZEVRIA at 43 days post-vaccination. (D) FACS example of SARS-CoV-2 spike-specific circulating follicular helper T (cTFH) cells (CXCR5 + OX40 + surface CD40L+, as a percentage of CD4+ T cells) after stimulation with spike MP. (E) Frequencies of SARS-CoV-2 spike-specific cTFH cells induced by COVIVAC at 3 μg and 6 μg and VAXZEVRIA at day 1 (baseline) and at day 43 post-vaccination. (F) Comparison of spike-specific cTFH cells induced by COVIVAC at 3 μg and 6 μg and VAXZEVRIA at 43 days post-vaccination. Dotted green lines indicate the limit of quantification (LOQ). Light gray, COVIVAC at 3 μg; red, COVIVAC at 6 μg; black, VAXZEVRIA. G) Correlation of spike specific CD4 T cell responses and neutralising antibody titers measured 43 days post-vaccination for COVIVAC at 3 μg (gray line) and 6 μg (red line) and VAXZEVRIA (black line). H) Comparison of spike specific CD8+ T cells induced by COVIVAC at 3 μg and 6 μg and VAXZEVRIA at 43 days post-vaccination. I) Comparison of spike specific CD8+ T cells s induced by COVIVAC at 3 μg and 6 μg and VAXZEVRIA at 43 days post-vaccination. The bars in (B, C, E, F, H, I) indicate the geometric mean and geometric SD in the analysis of the spike-specific T cell frequencies. Data were analysed for statistical significance using the Mann-Whitney U test (B, C, E, F, H, I). Background- subtracted and log data analysed in all cases. (For interpretation of the references to colour in this figure legend, the reader is referred to the web version of this article.)

Finally, we explored the induction of spike specific CD4+ T cell responses by COVIVAC and VAXZEVRIA in a random subset of vaccinated individuals with no detectable anti-spike IgG by ELISA at baseline. Spike specific CD4+ T cell response was assessed utilizing an activation-induced molecules (AIM) assay, which evaluates the frequency of antigen-specific T cells based on the co-expression of OX40 and CD137 for CD4+ T cells and CD69 and CD137 of CD8 + T cells (Fig. 3). We detected induction of a spike specific CD4+ T cell response on day 43 in all of 10 COVIVAC 3 μg vaccinees with a 0·14 % cell frequency (95 % CI 0·074–0·27 %), in 9 of 10 COVIVAC 6 μg vaccinees with a 0·092 % cell frequency (95 % CI 0·040–0·21 %), and in all of 12 VAXZEVRIA vaccinees with a 0·18 % cell frequency (95 % CI 0·095–0·34 %) (Fig. 3B). The intensity of spike specific CD4+ T cell induction on day 43 was similar among the treatment groups (Fig. 3C).

Follicular helper T (T_FH_) cells help B cells activate antibody production. As this T cell subset can be induced by SARS-CoV-2 infection and COVID-19 vaccination, we evaluated the frequency of circulating T_FH_ cells by the AIM assay at baseline (day 1) and post-dose two (day 43) (Fig. 3E). cT_FH_ were detected on day 43 in 8 of 10 COVIVAC 3 μg vaccinees with a 0·077 % cell frequency (95 % CI 0·020–0·097 %), in 4 of 10 COVIVAC 6 μg vaccinees with a 0·044 % cell frequency (95 % CI 0·041–0·14 %), and in 9 of 12 VAXZEVRIA vaccinees with a 0.08 % cell frequency (95 % CI 0·040–0·16 %) (Fig. 3E). As shown for the spike specific CD4+ T cells, the intensity of spike specific cT_FH_ cells induction on day 43 was similar among treatment groups (Fig. 3F).

Antibody levels by PNA and frequencies of memory CD4+ T cells were significantly correlated for COVIVAC 3 μg (*r* = 0.824, *p* > 0.0001), COVIVAC 6 μg (*r* = 0.875, p > 0.0001), and VAXZEVRIA (*r* = 0.764, p > 0.0001) (Fig. 3G); this finding is evidence of a coordinated cellular-humoral immune response in both COVIVAC and VAXZEVRIA recipients.

Spike specific CD8+ T cells were also measured by AIM (CD69+ CD137+). We detected a response on day 43 in 1 of 10 COVIVAC 3 μg vaccinees with a 0·032 % cell frequency (95 % CI 0·0.028–0·0.036 %), in 1 of 10 COVIVAC 6 μg vaccinees with a 0·037 % cell frequency (95 % CI 0.023–0.052 %), and in 3 of 11 VAXZEVRIA vaccinees with a 0·049 % cell frequency (95 % CI 0.029–0.069 %) (Fig. 3H). The intensity of CD8+ T cell responses detected on day 43 was low and similar among the treatment groups. (Fig. 3I).

## 4 Discussion

This phase 2 study showed that COVIVAC (NDV-HXP-S), when administered as a two-dose series to adults, including those 60 years of age and older, has an acceptable safety profile. It is highly immunogenic, activating T cell responses, and eliciting neutralising antibody responses 14 days after vaccine dose two that are superior to those induced by the adenovirus vectored VAXZEVRIA comparator vaccine.

All treatments evaluated were well tolerated with predominantly mild and self-limited reactogenicity that was greater after dose one than after dose two. The COVIVAC formulations at 3 and 6 μg dose levels were less reactogenic after dose one than the VAXZEVRIA comparator with respect to self-reported pain at the injection site, myalgia, and incidence of fever. Otherwise, there were no notable differences. Overall, in this study of 374 participants, there were no spontaneously reported AEs attributed by investigators to vaccination and no concerns expressed by the DSMB providing safety oversight.

In terms of neutralising antibody titers, measured in a PNA, both dose levels of COVIVAC showed superiority to VAXZEVRIA within each age stratum at an early time point (14 days after dose two) with the trend continuing out to month 6, even though statistical significance was not reached at the later time point. Superior induction of neutralising antibody by COVIVAC at both dose levels relative to VAXZEVRIA 14 days after vaccine dose two was confirmed by exploratory testing using a live virus neutralisation assay. Interestingly, spike-binding antibodies were lower in the COVIVAC groups compared to the VAXZEVRIA group, hinting at a better ratio of neutralising to binding antibodies for COVIVAC. In fact, it has been shown in an earlier study, that inactivated NDV-HXP-S vaccines induce better ratios of neutralising antibodies relative to spike binding IgG compared to mRNA vaccines [19].

These findings are important since neutralising antibodies to SARS-CoV-2 are a mechanistic correlate of protection [5,20] and new SARS-CoV-2 vaccines (e.g. Corbevax and VLA2001) have been licensed based on immune-bridging of neutralising antibody titers [21,22]. Our results suggest that the possibility could be open for COVIVAC or for similar NDV-based vaccines to be developed by other manufacturers.

There was no improvement in the induction of vaccine homologous neutralising antibodies by doubling the dose from a 3 to 6 μg level. Considering the important dose effect on immunogenicity observed in the phase 1 trial comparing 10 and 3 μg dose levels, with no adverse impact on reactogenicity, further development of COVIVAC will likely revert to a 10 μg dose level [8].

In this comparative study, spike specific CD4+ T cell responses were detected in 90–100 % of a small subset of randomly selected individuals, all being seronegative for anti-spike IgG pre-vaccination, in test and comparator vaccine groups. This is comparable to what has been reported for other COVID-19 vaccines such as mRNA and adenovirus vector vaccines [14]. Similarly, we have detected circulating T follicular helper cells in a substantial fraction of vaccinees, supported by the strong correlation of spike-specific CD4+ T cell responses and functional antibody responses. In previous studies, we demonstrated that a coordinated cellular-humoral immune response is associated with mild disease outcomes in infected individuals.^14,18.^

This study has several limitations. First, it was a phase 2 trial of limited size with no clinical endpoint. Second, the investigational and comparator vaccines expressed an ancestral spike immunogen. Moreover, the study population was largely naïve to SARS-CoV-2 at the time they were vaccinated. Currently, COVID-19 vaccines are being deployed for booster immunization in primed but at-risk adults. While COVIVAC performed well by inducing neutralising antibodies, its use as a booster vaccine is yet to be evaluated. Vaccines with ancestral spike antigens are obsolete now due to emergence of different variants, especially the Omicron variant family. Current recommendations from regulatory authorities and WHO state that XBB-lineage spike antigens should be used in updated vaccines. GMP seed viruses for COVIVAC with XBB.1.5 spike exist and can be used for manufacturing of strain-changed updated vaccines. We did not evaluate induction of neutralising antibodies to vaccine heterologous variants, as this was outside of the scope of this study. Nevertheless, we observed that COVIVAC induced CD4+ T cell responses comparable to the VAXZEVRIA comparator, and it has been reported that CD4+ T cell responses induced by the ancestral spike protein are maintained and cross-recognize SARS-CoV-2 variants, from Alpha to Omicron [23,24].

The study was conducted during the COVID-19 pandemic, thus exposure to intercurrent SARS-CoV-2 (both subclinical and clinically overt), especially between study days 43 and 197 was both inevitable and expected to be randomly distributed among the three study arms. The study was not designed to identify suspected or confirmed COVID-19 episodes. We acknowledge that these environmental exposures confound the immunogenicity assessment done on day 197.

Strengths of this study are the use of a fully validated functional antibody readout (PNA), the inclusion of older adults with an age-stratified analysis showing preservation of immunogenicity despite increased age, the assessment of T cell responses, and the selection of the VAXZEVRIA vaccine as a highly relevant immuno-bridging comparator. The efficacy of the VAXZEVRIA vaccine has been demonstrated in multiple double-blind randomised clinical trials, varying from approximately 70 % against any symptomatic disease to >95 % against severe disease and/or hospitalization [25]. Multiple effectiveness and observational studies confirmed the high level of protection afforded by the vaccine, leading to its approval in the UK and other European countries [26]. By early 2022, the VAXZEVRIA vaccine had been approved by over 170 countries, including Vietnam, making it the most widely deployed vaccine across the globe with over 2.5 billion doses used [26]. The induction of superior levels of neutralising antibodies by COVIVAC and similar activation of CD4+ T cells in comparison to VAXZEVRIA strongly suggest that COVIVAC's effectiveness would be at least similar.

The CD4+ and CD8+ T cell response has been assessed using the AIM assay measuring the frequency of spike-specific T cell responses. It is important to point out that functional capacity of T cell responses, such as through production of cytokines, need to be assessed for a comprehensive picture of vaccine-induced spike-specific T cell responses [27].

The clinical trial was designed to assess the feasibility of conducting a phase 3 trial in which the benefit of vaccination with COVIVAC could be confirmed by demonstrating non-inferior or superior immunogenicity relative to an authorized comparator COVID-19 vaccine. That aim was met. Further development of COVIVAC updated to express a contemporary recombinant spike protein, administered as a booster dose to vulnerable individuals, is a viable option for its manufacturer IVAC, which serves the public sector of Vietnam.

## **Funding**

Vietnam's Institute of Vaccines and Medical Biologicals (including support from Vietnam's national COVID-19 vaccine fund and a charitable contribution from the Thien Tam fund of Vin group), Coalition for Epidemic Preparedness Innovations, a charitable contribution from 10.13039/100004326Bayer AG, US National Institutes of Health

## CRediT authorship contribution statement

**Vu Dinh Thiem:** Writing – review & editing, Investigation, Data curation. **Dang Duc Anh:** Writing – review & editing, Supervision, Resources, Investigation, Data curation. **Vu Hai Ha:** Writing – review & editing, Investigation, Data curation. **Nguyen Van Thom:** Writing – review & editing, Resources, Investigation, Data curation. **Tran Cong Thang:** Writing – review & editing, Project administration, Data curation, Conceptualization. **Jose Mateus:** Writing – review & editing, Supervision, Investigation, Data curation. **Juan Manuel Carreño:** Writing – review & editing, Supervision, Investigation, Data curation. **Rama Raghunandan:** Writing – original draft, Project administration, Methodology, Funding acquisition. **Nguyen Mai Huong:** Writing – review & editing, Project administration, Data curation, Conceptualization. **Laina D. Mercer:** Writing – review & editing, Validation, Methodology, Formal analysis, Data curation, Conceptualization. **Jorge Flores:** Writing – review & editing, Methodology, Data curation. **E. Alexandar Escarrega:** Writing – review & editing, Investigation. **Ariel Raskin:** Writing – review & editing, Investigation. **Duong Huu Thai:** Writing – review & editing, Project administration, Data curation, Conceptualization. **Le Van Be:** Writing – review & editing, Project administration, Funding acquisition, Conceptualization. **Alessandro Sette:** Writing – review & editing, Resources, Funding acquisition. **Bruce L. Innis:** Writing – original draft, Visualization, Supervision, Funding acquisition, Conceptualization. **Florian Krammer:** Writing – original draft, Supervision, Resources, Methodology, Funding acquisition, Conceptualization. **Daniela Weiskopf:** Writing – original draft, Supervision, Resources, Methodology, Investigation, Funding acquisition, Data curation, Conceptualization.

## Declaration of competing interest

The authors declare the following financial interests/personal relationships which may be considered as potential competing interests: Alessandro Sette reports financial support was provided by National Institutes of Health. Daniela Weiskopf reports a relationship with Moderna Inc. that includes: consulting or advisory. Daniela Weiskopf is an associate editor for Vaccine. La Jolla Institute has patents issued for various aspects of T cell epitope and vaccine design work. Juan Manuel Carreno reports funding was provided by Icahn School of Medicine at Mount Sinai Department of Microbiology. Florian Krammer reports a relationship with Avimex Laboratories that includes: royalties or licenses and consulting. Florian Krammer reports a relationship with Kantaro Biosciences that includes: royalties or licenses. Florian Krammer reports a past relationship with Pfizer that includes: consulting or advisory. Florian Krammer reports a past relationship with Seqirus Inc. that includes: consulting or advisory. Florian Krammer reports a past relationship with GSK that includes: consulting or advisory. Florian Krammer reports a relationship with Gritstone bio Inc. that includes: consulting or advisory. Florian Krammer reports a relationship with CastleVax that includes: equity or stocks, royalties or licenses, and consulting. Florian Krammer has patents regarding SARS-CoV-2 vaccines and serological assays issued to Icahn School of Medicine at Mount Sinai. All other authors declare that they have no known competing financial interests or personal relationships that could have appeared to influence the work reported in this paper.

## Data Availability

Deidentified participant data will be made available for two years after publication upon request directed to the lead author

## Appendix A Supplementary data

Supplementary material 1 Image 1

**Supplementary material 2: Fig. S1: Gating strategy to identify CD4 and CD8 T lymphocytes:** Representative gating strategy to define CD3 + CD4+ and CD3 + CD8+ cells by AIM assay. Image 2
