# Supplementary material for: Safety and immunogenicity of an inactivated recombinant Newcastle disease virus vaccine expressing SARS-CoV-2 spike: A randomised, comparator-controlled, phase 2 trial
Source: Vaccine. 2025 Jan 12;44:None. doi: 10.1016/j.vaccine.2024.126542 (PMC11672239; doi:10.1016/j.vaccine.2024.126542)
Supplement: Supplementary file 1 — Supplementary material 1 [file mmc1.pdf]

Supplemental Table 1: Antibodies used in the AIM assay

Reagent	Clone (Source)	Catalog No	Dilution
CD40 pure- functional grade	HB14 (Miltenyi Biotec)	130-094-133	1.5:100
CCR7 - BV711	G043H7 (Biolegend)	353228	1:200
Fixable Live/Dead Blue	(Thermo)	L23105	1:1000
Human FC Block	(BD)	564220	1:20
CD3 - BUV395	UCHT1 (BD)	563546	1:1000
CD8 - BUV805	SK1 (BD)	612889	1:1000
CD16 - BV510	3G8 (Biolegend)	302048	1:1000
CD14 - BV510	63D3 (Biolegend)	367124	1:1000
CD20 - BV510	2H7 (Biolegend)	302340	1:1000
CD45RA - BV570	HI100 (Biolegend)	304132	1:1000
CD4 - cFluor b548	SK3 (Cytek)	R7-20043	1:500
PD-1 - BV785	EH12.2H7 (Biolegend)	329930	1:200
CD69 - FITC	FN50 (Biolegend)	310904	1:200
CD40L - PE-Dazzle594	24-31 (Biolegend)	310840	1:200
CD137 - BUV737	4b4-1 (BD)	741861	1:100
OX40 - APC	Ber-Act35 (Biolegend)	350008	1:100
Brilliant Staining Buffer Plus	(BD)	566385	1:10

Supplement Table 2: Number of subjects with unsolicited adverse events with onset during 28 days after vaccination in the safety analysis population

	COVIVAC		
**Number of subjects with:**	**3 µg (N = 124)** n (%) (95% CI*)	**6 µg (N = 125)** n (%) (95% CI*)	**VAXZEVRIA (N = 125)** n (%) (95% CI*)
**One or more adverse events**	n= 124	n= 125	n= 125
	36 (29·0%)	29 (23·2%)	39 (31·2%)
	(21·2-37·9)	(16·1-31·6)	(23·2-40·1)
**Vaccine-related adverse events**	n= 124	n= 125	n= 125
	0 (0%)	0 (0%)	0 (0%)
	(0·0-2·9)	(0·0-2·9)	(0·0-2·9)
**Withdrawn due to an adverse event**	n= 124	n= 125	n= 125
	0 (0%)	0 (0%)	0 (0%)
	(0·0-2·9)	(0·0-2·9)	(0·0-2·9)

Note: Two-sided 95% confidence intervals (CIs) computed via the Clopper-Pearson method.

Supplement Table 3: Summary of geometric mean titer (GMT) of NT_{50} against SARS-CoV-2 pseudovirus by age group in the full analysis population

NT_{50} measure			COVIVAC 3 µg (N = 124)	COVIVAC 6 µg (N = 125)	VAXZEVRIA (N = 125)
Baseline (D1)	18-59 yr.	GMT (95% CI)	n = 80	n = 79	n = 79
			5·04 (4·96 , 5·13)	5·00 (-)	5·23 (4·78 , 5·72)
	≥ 60 yr.	GMT (95% CI)	n = 40	n = 41	n = 34
			5·00 (-)	5·14 (4·86 , 5·45)	5·12 (4·88 , 5·36)
14 days after the second vaccination (D43)	18-59 yr.	GMT (95% CI)	n = 80	n = 78	n = 79
			153·28 (124·22 , 189·15)	176·52 (141·45 , 220·27)	99·92 (80·80 , 123·56)
	≥ 60 yr.	GMT (95% CI)	n = 40	n = 40	n = 35
			183·57 (133·40 , 252·61)	257·87 (181·10 , 367·18)	79·49 (55·68 , 113·46)
6 months after the second vaccination (D197)	18-59 yr.	GMT (95% CI)	n = 78	n = 78	n = 77
			188·05 (100·93 , 350·38)	140·99 (76·57 , 259·61)	126·74 (77·40 , 207·55)
	≥ 60 yr.	GMT (95% CI)	n = 38	n = 37	n = 34
			98·94 (40·18 , 243·62)	116·19 (47·80 , 282·45)	50·00 (21·18 , 118·03)

Note: - NT_{50} GMTs are analyzed from subjects with seronegative anti-S IgG at baseline.

Supplement Table 4: Percentage of subjects with NT_{50} titer sero responses against SARS-CoV-2 pseudovirus as defined by a ≥ 4-fold increase from baseline by age group in the full analysis population

NT_{50} measure ≥ 4-fold (Titer)			COVIVAC 3 µg (N = 124)	COVIVAC 6 µg (N = 125)	VAXZEVRIA (N = 125)
14 days after the second vaccination (D43)	18-59 yr.	n (%) (95% CI)	n = 82	n = 83	n = 82
			73 (89·0%)	77 (92·8%)	70 (85·4%)
			(80·2-94·9)	(84·9-97·3)	(75·8-92·2)
	≥ 60 yr.	n (%) (95% CI)	n = 42	n = 40	n = 40
			41 (97·6%)	38 (95·0%)	30 (75·0%)
			(87·4-99·9)	(83·1-99·4)	(58·8-87·3)
6 months after the second vaccination (D197)	18-59 yr.	n (%) (95% CI)	n = 80	n = 82	n = 80
			44 (55·0%)	39 (47·6%)	44 (55·0%)
			(43·5-66·2)	(36·4-58·9)	(43·5-66·2)
	≥ 60 yr.	n (%) (95% CI)	n = 40	n = 37	n = 37
			17 (42·5%)	17 (45·9%)	13 (35·1%)
			(27·0-59·1)	(29·5-63·1)	(20·2-52·5)

Note: The 95% CIs are computed via the Clopper-Pearson method. The analysis included subjects regardless of anti S IgG status at baseline.

Supplement Table 5: Summary of geometric mean titer ratio (GMT Ratio) of NT_{50} against SARS-CoV-2 pseudovirus by age group in the full analysis population

NT_{50} measure GMT ratio (95% CI)		COVIVAC		
		**6 µg / 3 µg**	**3 µg / VAXZEVRIA**	**6 µg / VAXZEVRIA**
Baseline (D1)	18-59 yr.	0·99 (0·97 , 1·01)	0·96 (0·88 , 1·05)	0·96 (0·88 , 1·04)
	≥ 60 yr.	1·03 (0·97 , 1·09)	0·98 (0·94 , 1·02)	1·01 (0·93 , 1·08)
14 days after the second vaccination (D43)	18-59 yr.	1·15 (0·85 , 1·56)	1·53 (1·14 , 2·06)	1·77 (1·30 , 2·40)
	≥ 60 yr.	1·40 (0·88 , 2·24)	2·31 (1·45 , 3·69)	3·24 (1·98 , 5·32)
6 months after the second vaccination (D197)	18-59 yr.	0·75 (0·32 , 1·78)	1·48 (0·67 , 3·27)	1·11 (0·51 , 2·43)
	≥ 60 yr.	1·17 (0·34 , 4·08)	1·98 (0·58 , 6·77)	2·32 (0·69 , 7·85)

Note: NT_{50} GMTs are analyzed from subjects with seronegative anti-S IgG at baseline.

Supplement Table 6: GMT of NT_{50} against vaccine-homologous SARS-CoV-2 pseudovirus (PNA) compared to live virus (LVNA) 14 days after the second vaccination (D43), with GMT ratio contrasting COVIVAC to AZ in the full analysis population

**NT_{50} by PNA**			
**Statistic**	**COVIVAC 3**	**COVIVAC 6**	**VAXZEVRIA**
N	120	118	114
GMT	162.78	200.72	93.14
95% CI	(136.79, 193.71)	(166.15, 242.47)	(77.70, 111.66)

**Statistic**	**COVIVAC 3/VAXZEVRIA**	**COVIVAC 6/VAXZEVRIA**
GMT ratio	1.75	2.15
95% CI	(1.36, 2.24)	(1.66, 2.80)

**NT_{50} by LVNA**			
**Statistic**	**COVIVAC 3**	**COVIVAC 6**	**VAXZEVRIA**
N	120	117	114
GMT	45.84	70.35	29.84
95% CI	(35.82, 58.66)	(55.91, 88.53)	(23.74, 37.52)

**Statistic**	**COVIVAC 3/VAXZEVRIA**	**COVIVAC 6/VAXZEVRIA**
GMT ratio	1.54	2.36
95% CI	(1.10, 2.15)	(1.71, 3.26)

Note: NT_{50} GMTs are analyzed from subjects with seronegative anti-S IgG at baseline.

Supplement Table 7: Summary of geometric mean concentration (GMC) in BAU/mL of anti-S IgG assessed by ELISA by age group in the full analysis population

Anti-S IgG measure (BAU/mL)			COVIVAC 3 µg (N = 124)	COVIVAC 6 µg (N = 125)	VAXZEVRIA (N = 125)
Baseline (D1)	18-59 yr.	GMC (95% CI)	n = 80	n = 79	n = 80
			3.15 (-)	3·15 (-)	3·15 (-)
	≥ 60 yr.	GMC (95% CI)	n = 40	n = 41	n = 35
			3·15 (-)	3·15 (-)	3·15 (-)
14 days after the second vaccination (D43)	18-59 yr.	GMC (95% CI)	n = 80	n = 78	n = 79
			139·60 (112·48 , 173·27)	155·46 (123·39 , 195·87)	366·41 (306·59 , 437·91)
	≥ 60 yr.	GMC (95% CI)	n = 40	n = 40	n = 35
			146·65 (104·62 , 205·56)	224·91 (155·86 , 324·57)	312·37 (211·23 , 461·93)
6 months after the second vaccination (D197)	18-59 yr.	GMC (95% CI)	n = 78	n = 78	n = 77
			166·99 (87·31 , 319·39)	124·64 (65·78 , 236·19)	277·29 (164·97 , 466·07)
	≥ 60 yr.	GMC (95% CI)	n = 40	n = 39	n = 34
			78·28 (30·39 , 201·61)	89·87 (35·08 , 230·22)	107·22 (42·89 , 268·03)

Note: GMCs are analyzed from subjects with seronegative anti-S IgG at baseline.

Supplement Table 8: Summary of geometric mean concentration ratio (GMC Ratio) of anti-S IgG assessed by ELISA by age group in the full analysis population

Anti-S IgG GMC ratio (95% CI)		COVIVAC		
		**6 µg / 3 µg**	**3 µg / VAXZEVRIA**	**6 µg / VAXZEVRIA**
Baseline (D1)	18-59 yr.	1·00 (1·00 , 1·00)	1·00 (1·00 , 1·00)	1·00 (1·00 , 1·00)
	≥ 60 yr.	1·00 (1·00 , 1·00)	1·00 (1·00 , 1·00)	1·00 (1·00 , 1·00)
14 days after the second vaccination (D43)	18-59 yr.	1·11 (0·81 , 1·52)	0·38 (0·29 , 0·50)	0·42 (0·32 , 0·57)
	≥ 60 yr.	1·53 (0·94 , 2·51)	0·47 (0·28 , 0·78)	0·72 (0·42 , 1·22)
6 months after the second vaccination (D197)	18-59 yr.	0·75 (0·30 , 1·84)	0·60 (0·26 , 1·37)	0·45 (0·20 , 1·02)
	≥ 60 yr.	1·15 (0·31 , 4·27)	0·73 (0·20 , 2·70)	0·84 (0·23 , 3·07)

Note: Anti-S IgG GMCs ratio are analyzed from subjects with seronegative anti-S IgG at baseline.

Supplement Table 9: Summary of geometric mean fold rise (GMFR) from baseline of anti-S IgG concentration assessed by ELISA by age group in the full analysis population

Anti-S IgG measure			COVIVAC 3 µg (N = 124)	COVIVAC 6 µg (N = 125)	VAXZEVRIA (N = 125)
14 days after the second vaccination (D43)	18-59 yr.	GMFR from baseline (95% CI)	n = 81	n = 82	n = 83
			45·18 (36·37 , 56·12)	46·55 (37·08 , 58·43)	108·47 (89·94 , 130·83)
	≥ 60 yr.	GMFR from baseline (95% CI)	n = 42	n = 41	n = 41
			45·03 (32·46 , 62·45)	69·32 (48·25 , 99·59)	87·13 (60·32 , 125·84)
6 months after the second vaccination (D197)	18-59 yr.	GMFR from baseline (95% CI)	n = 79	n = 83	n = 81
			54·93 (28·85 , 104·60)	39·87 (21·68 , 73·30)	78·74 (47·13 , 131·54)
	≥ 60 yr.	GMFR from baseline (95% CI)	n = 42	n = 39	n = 40
			22·64 (9·12 , 56·18)	28·52 (11·13 , 73·06)	35·38 (15·15 , 82·63)

Note: Geometric Mean Fold Rise (GMFR) is the geometric mean of the ratios of post-vaccination to the pre-first vaccination at D1 (baseline).
