# Supplementary figures and images for: Safety and immunogenicity of an inactivated recombinant Newcastle disease virus vaccine expressing SARS-CoV-2 spike: A randomised, comparator-controlled, phase 2 trial
Source: Vaccine. 2025 Jan 12;44:None. doi: 10.1016/j.vaccine.2024.126542 (PMC11672239; doi:10.1016/j.vaccine.2024.126542)

Supplement figure S1: Gating strategy

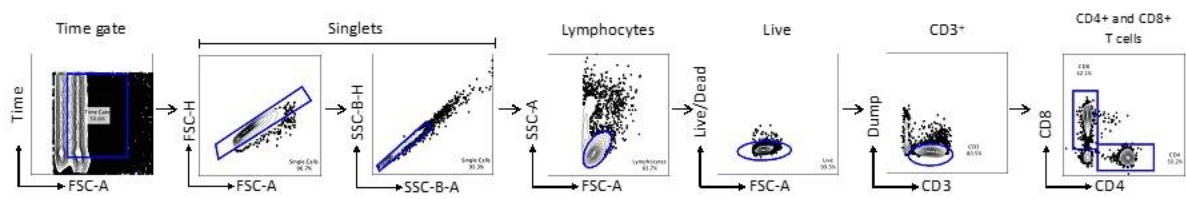

Supplement: Supplementary file 2 — Supplementary material 2: Fig. S1: Gating strategy to identify CD4 and CD8 T lymphocytes: Representative gating strategy to define CD3 + CD4+ and CD3 + CD8+ cells by AIM assay. [file mmc2.pdf]
